# FaIRClocks: Fair and Interpretable Representation of the Clock Drawing Test for mitigating classifier bias against lower educational groups

**DOI:** 10.21203/rs.3.rs-3398970/v1

**Published:** 2023-10-09

**Authors:** Jiaqing Zhang, Sabyasachi Bandyopadhyay, Faith Kimmet, Jack Wittmayer, Kia Khezeli, David J. Libon, Catherine C. Price, Parisa Rashidi

**Affiliations:** 1 Department of Electrical and Computer Engineering, University of Florida; 2 J. Crayton Pruitt Family Department of Biomedical Engineering, University of Florida; 3 Department of Computer and Information Science and Engineering, University of Florida; 3 Department of Clinical and Health Psychology, College of Public Health and Health Professions, University of Florida; 4 Department of Anesthesiology, College of Medicine, University of Florida; 5 Perioperative Cognitive Anesthesia Network^SM^, University of Florida; 6 Department of Geriatrics and Gerontology, Department of Psychology, New Jersey Institute for Successful Aging, School of Osteopathic Medicine, Rowan University; 7 Intelligent Critical Care Center (IC3), University of Florida.

**Keywords:** AI Fairness, semi-supervised deep learning, Relevance Factor Variational Autoencoder, Mini-mental state examination, attention, memory

## Abstract

The clock drawing test (CDT) is a neuropsychological assessment tool to evaluate a patient’s cognitive ability. In this study, we developed a **Fa**ir and **I**nterpretable **R**epresentation of **Clock** drawing tests (**FaIRClocks**) to evaluate and mitigate bias against people with lower education while predicting their cognitive status. We represented clock drawings with a 10-dimensional latent embedding using Relevance Factor Variational Autoencoder (RF-VAE) network pretrained on publicly available clock drawings from the National Health and Aging Trends Study (NHATS) dataset. These embeddings were later fine-tuned for predicting three cognitive scores: the Mini-Mental State Examination (MMSE) total score, attention composite z-score (ATT-C), and memory composite z-score (MEM-C). The classifiers were initially tested to see their relative performance in patients with low education (<= 8 years) versus patients with higher education (> 8 years). Results indicated that the initial unweighted classifiers confounded lower education with cognitive impairment, resulting in a 100% type I error rate for this group. Thereby, the samples were re-weighted using multiple fairness metrics to achieve balanced performance. In summary, we report the FaIRClocks model, which a) can identify attention and memory deficits using clock drawings and b) exhibits identical performance between people with higher and lower education levels.

## Introduction

Cognitive impairment is a growing concern in today’s aging population. Today, more than 6 million Americans of all ages are living with Alzheimer’s disease^1^. Alzheimer’s is one of the leading causes of death in the US, costing more than 345 billion dollars yearly for treatment^2^. Therefore, early detection of cognitive impairment and constant monitoring of cognitive health in older adults is an essential area of interest. The clock drawing test (CDT) is a widely recognized and simple cognitive screening tool used to evaluate different aspects of cognitive function, including attention, memory, and executive function^3,4^. The CDT consists of two parts: a) the *command* test condition, where the patient is required to “draw the face of a clock, put in all the numbers, and set the hands to *ten after eleven*,” followed by b) the *copy* test condition, where the patient is required to copy a model clock^4^. The command condition assesses a person’s ability to understand and execute three commands requiring comprehension, working memory, semantic knowledge, inhibitory function, visuoconstruction, and planning abilities^4,5^. In contrast, the copy condition primarily assesses visuospatial skills and planning^4–6^. The combination of these two conditions has proven to be beneficial in differential dementia diagnoses.^4,6,7^

Years of formal education play an essential role in premorbid intellect and cognitive reserve, as education can protect against later cognitive decline, including protection from neurological insult and neurodegenerationp^8–11^. Furthermore, total years of formal education is a predictor of postoperative cognitive decline and postoperative hospital visits^12–14^. Appreciating this fact is relevant to the care of older patients, as census data show they have more frequently fewer years of formal education relative to younger generations^15,16^.

For individuals with less than eight years of education, cognitive screening tests such as the MiniCog, which includes clock drawing, suffer from an inflated type I error in predicting dementia^17^. Previous literature indicates that CDT performance is affected by age, education level, race, and ethnicity^18^. A systematic review showed that higher levels of education positively affected clock drawing performance, while illiteracy hindered clock construction in several studies^19^. Specifically, individuals with fewer years of education tend to exhibit more digit placement errors and increased omission of clock hands^20,21^. In this study, we have investigated the effect of education and racial bias in cognitive classifiers developed on newly published deep learning (DL) representations of clock drawing^22^. We used state-of-the-art bias mitigation algorithms to identify and mitigate educational and racial bias in classifiers constructed on these deep CDT representations.

In recent years, machine learning, especially deep learning, has become a burgeoning tool in cognitive health research. The deep convolutional neural network (CNN) has proved effective for scoring CDT^23,24^. A few studies have also used a segmentation CNN model to extract sub-features from the clock drawing, including clockface, digits, and hands, and another CNN model to score the segmented outcomes separately^25,26^. However, these models require large, high-quality annotated clock drawing datasets, which are time- and labor-intensive to obtain. Additionally, the black-box nature of DL models curtails their interpretability. With the rapid advancement of computer vision, self-supervised deep generative models can be trained to create deep representations of clock drawings, which are robust, interpretable, and broadly applicable to different downstream classification tasks. Previously, we have proposed a semi-supervised variational autoencoder (VAE) method to represent clock drawings using a compressed two-dimensional feature vector. This representation was encoded within an interpretable two-dimensional latent space that could classify clocks from patients with and without dementia with moderate performance^27^. In another study, we utilized the Relevance Factor Variational Autoencoder (RF-VAE)^28^ model to discover a complete set of graphomotor anomalies in clock drawings. This significantly improved the performance of classifying dementia patients^22^.

We followed our prior work^22^ in this study by pretraining an RF-VAE model on a large, unlabeled, publicly available dataset from the National Health and Ageing Trends Study (NHATS) and fine-tuning the model on the dataset collected by the University of Florida and Shands Hospital as part of a federal investigation. We created Fair and Interpretable Representations of CDT (FaIRClocks) for rarely studied cognitive outcomes such as attention and memory deficits. Specifically, we created three different classifiers for predicting the Mini-Mental State Examination (MMSE) total score, attention composite (ATT-C), and memory composite (MEM-C) Z-scores. We then analyzed the classifier biases in these models against people in under-represented demographics such as lower education (<= 8 years), Black, and Hispanic groups. We mitigated the classifiers’ bias using IBM’s AI Fairness Toolkit^29^ and Shapley Additive Explanations (SHAP) analysis^30^ to understand the change in the relative importance of the features in these classifiers before and after bias mitigation. The contributions of our work are summarized as follows:

1. We propose a novel, fair, and interpretable semi-supervised deep learning model for identifying multiple cognitive deficits with clock drawings, i.e., FAIRClocks.
2. We detect classifier bias against lower education level (less than 8 years of formal education) when predicting MMSE and ATT-C, but no educational bias was detected in MEM-C prediction. However, we detected racial bias in the MEM-C classifier.
3. With the help of AIF360 toolbox, we mitigate demographic biases from our classifiers, which significantly improved the sensitivity to specificity balance in these under-represented groups.

## Results

### Patients

Table 1 describes the demographic characteristics of patients who performed the CDT, divided into training and testing cohorts. The data were collected at the University of Florida (UF) and the UF Health Shands hospital as part of an NIH investigation using a honest data broker IRB-approved method. Out of the 863 patients enrolled in the study, neuropsychologists administered the MMSE test to 840 people, with ATT-C and MEM-C composites calculated for 731 people. Not every patient completed both the copy condition and the command conditions of the test. Specifically, one patient completed only the copy test; conversely, two completed only the command test. Detailed demographic information is in Table 1.

### RF-VAE based latent representation of the NHATS dataset

**Figure 1.**
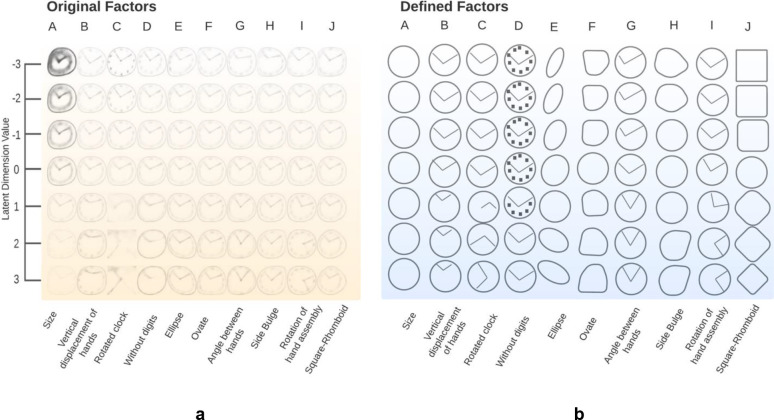
During the training phase, the RF-VAE model derives a 10D latent representation from the clock drawings in the NHATS dataset. (a) Displayed are the reconstructed clock drawings corresponding to each latent variable. Columns denote individual latent dimensions, ranging from −3 (top) to +3 (bottom). (b) Interpretations for each latent space traversal are provided. The distinct constructional attributes of the clock drawings, which are most representative of the traversal of reconstructed clocks across a specific latent dimension, serve to characterize each latent dimension.

Figure 1 shows the reconstruction of the ten disentangled features in the latent space learned by RF-VAE using the NHATS dataset. The X-axis shows our definition of the ten features, i.e., size, vertical displacement of hands, rotated clockface, loss of digits, ellipse, ovate, angle between hands, side bulge, rotation of hand assembly, and square-rhomboid clockface. The Y-axis is the value of latent variables, which spans a range from −3 to 3. Column A shows the change in the brightness of the decoded clock drawings, which reflects the size of the original clock drawings. This is because all clock drawings are resized to 64×64 pixels, causing the larger clocks to lose some textural information compared to the smaller clocks. Column B shows the vertical displacement of hands as the latent value increases. Column C shows the rotation of the clockface. This is an artifact of the presence of rotated clock images within the NHATS dataset. Column D indicates the loss of digits on the clock face as this latent dimension increases. Column E shows a change in the degree and the direction of the eccentricity of a clockface. Column F shows a shift in the degree and direction of the ovate/obovate (leaf-shaped) clockfaces. Column G shows a decrease in the angle between the clock hands as this latent dimension increases. Column H shows the existence of a vertical asymmetry or a side-bulge in the clockface. The degree and orientation of this bulge changes along this latent dimension. Column I show the rotation of the clock hands’ assembly. Increase of this latent dimension indicate a clockwise rotation in their hand assembly. Column J shows the existence of non-circular clocks. With higher values along this latent dimension, the clock shape tends to be rhomboid. In contrast, lower values result in a square shape. These describe the 10 latent features discovered by the RF-VAE network on the NHATS dataset.

An identical RF-VAE network was previously trained on a dataset comprising 23,521 clock drawings collected through the UF and the UF Health Shands hospital system. This RF-VAE described in Bandyopadhyay et.al.^22^ had converged upon a similar group of features, namely: size, obovate, prolate-oblate (i.e., flattened or elongated clockfaces), vertical displacement of hands, eccentricity of ellipsoidal clockfaces, angle between hands, square-rhomboid clockfaces, ellipsoidal clockfaces in a reversed direction, side bulge and rotation of the hand assembly. In our current study, the only additional feature we found is the dimension encoding a loss of digits on the clockface. However, this feature was encoded in a combined manner with the vertical displacement of hands in the previously published study^22^. In Supplementary Table 1, we have provided an analysis of performance comparisons between classifiers constructed using the previously published RF-VAE encoder and the one developed in this study.

### Classification performance of the FAIRClocks models

Table 2 and Table 3 show the performance of our proposed FAIRClocks model. As inputs, these classifiers take the 10D latent representation obtained by the RF-VAE network on the NHATS dataset and demographic information (age, education, sex, race, and ethnicity). Then, we used the fairness function to reweight the training contributions of clocks drawn by patients from under-represented demographic categories. We trained three classifiers to predict “cognitive impairment” as captured by the three cognitive measures: MMSE total score, ATT-C z-score, and MEM-C z-score separately. To investigate the differences between *command* and *copy* conditions of clock drawing, we conducted a set of three ablation studies: a) involving only command clocks, b) only copy clocks, and c) combination of command and copy clocks. We observed that training on the copy condition of clock drawing images creates the strongest classifier for MMSE; command and copy conditions create the strongest classifier for ATT-C, while command condition demonstrates superior performance in predicting the MEM-C outcome.

### Fairness analysis

**Figure 2.**
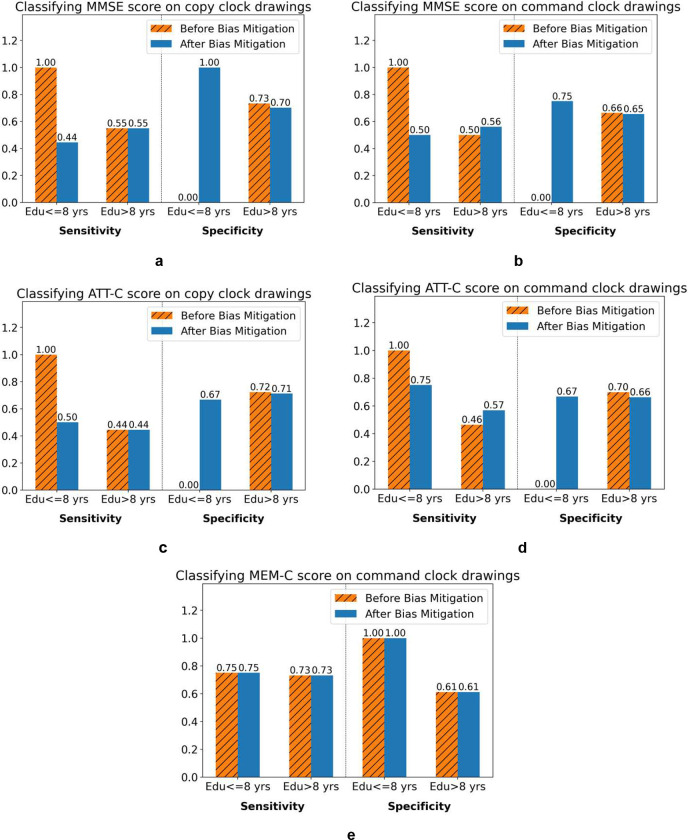
Effect of bias mitigation in classifying cognitive impairment by thresholding MMSE, ATT-C and MEM-C, in people with lower education (less than 8 years) versus those with higher education (greater than or equal to 8 years). Model sensitivity is shown in the left-side of each panel, and specificity is shown in the right-side of each panel. The orange bar with hatch (‘//’) shows the results before mitigating the bias (if it exists), and the blue bar shows the results after mitigating bias (if exists). (a) presents the model trained on the copy condition of clock drawings for predicting cognitive impairment from the MMSE score. (b) shows the results on command condition for predicting cognitive impairment from the MMSE score. (c) depicts the results on the copy condition for predicting cognitive impairment from the ATT-C. (d) illustrates the results of predicting ATT-C with command condition clock drawings as the only training data source. (e) reveals the situation where no bias was detected in the prediction based on MEM-C with command condition of the data which gives the best performance.

To measure the bias in model prediction on different demographic sub-groups, we evaluated the performance of the best model for each task on each of the subgroups, as shown in Table 2 and Table 3. We considered different demographic subgroups such as race: White/African-American, ethnicity: Hispanic/Non-Hispanic, and individuals with low education (less than or equal to 8 years of education) compared to those with high education (more than 8 years of education). We detected classification bias for patients with lower education in the task of MMSE and ATT-C classification. The “best” model predicted all clocks drawn by individuals with lower education to be characteristic of cognitive impairment as defined by the MMSE total score or ATT-C score. This was the case in both command and copy condition CDTs. This bias, depicted in Figure 2, represents a situation where the sensitivity value of the classifier for the low-education group is equal to 1, while its specificity value is equal to 0, thus creating a 100% type I error rate. We conducted a related samples t-test to test the difference in performance before and after reweighting the classifiers (shown in Supplementary Table 4). The p-values for both sensitivity and specificity suggest a significant difference in performance before and after bias mitigation through reweighting, observed in both the low-education and high-education groups. Bias related to education was not detected when predicting MEM-C, as shown in Figure 2(e). But, this model was unfair towards African-American patients compared with White patients in classifying “cognitive impairment” as defined by MEM-C scores, which is shown in Supplementary Table 2 and Supplementary Figure S1.

In general, overall classifiers, bias mitigation reduced performances by an average of 0.03 on area under the receiver operating curve (AUROC) for each task, shown in Table 2 and Table 3 (check-marked row), while the fairness metrics: 1-min(DI, 1/DI), and average odds different value decreased. For the low-education cohort, after bias removal, the balance between sensitivity and specificity improved significantly, as shown by the bar plots in blue color in Figure 2. Bias removal did not significantly impact the sensitivity and specificity balance in the high-education sub-group as can be seen in the orange bar with hatch (‘//’), and is further indicated by t-test results shown in Supplementary Table 4. These results are common across MMSE and ATT-C classification.

**Figure 3.**
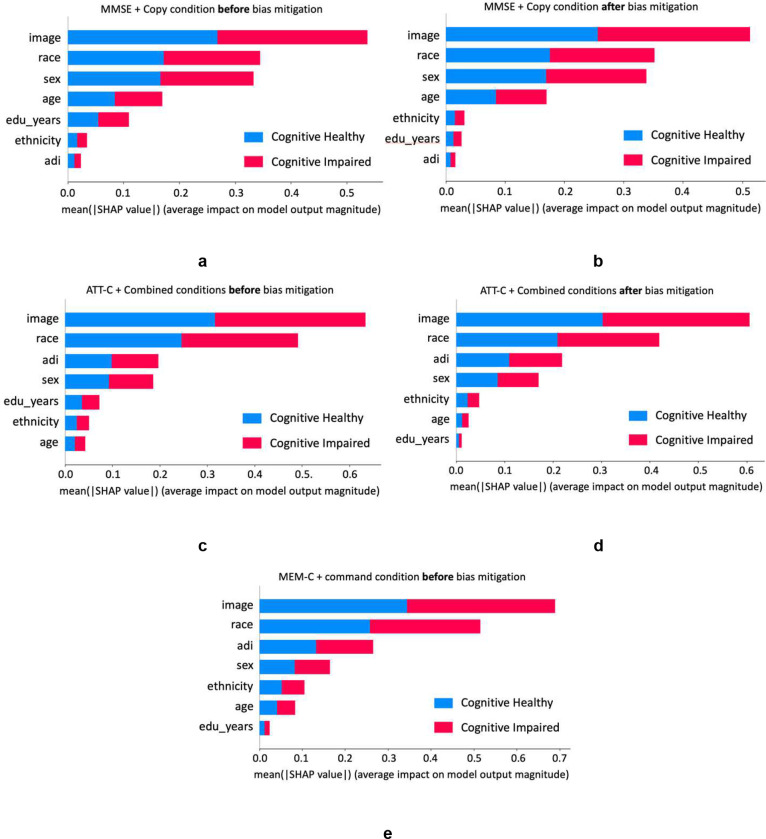
SHAP analysis of experiments on cognitive impairment prediction based on MMSE score using clock drawings under copy condition. The analysis includes the effect of both before and after applying a reweighting method. The y-axis represents the rank order of various features, while the x-axis quantifies the impact of each feature, with larger values indicating greater contributions. The “image” in the y-axis represents the 10-D representation of the clock drawings generated by the RF-VAE encoder. **a)** depicts the SHAP analysis prior to the application of the reweighting method for the best model in the MMSE setup. **b)** illustrates the resultant impact following the application of the reweighting method. Notably, the contribution made by the education level feature was tempered after reweighting was applied. **c)** shows the plot for the cognitive impairment prediction based on ATT-C before reweighting method. **d)** shows that after the reweighting function for ATT-C setup. **e)** highlights the results of the MEM-C setup. It only shows the result without the reweighting method applied because there is no bias detected.

The SHAP analysis shown in Figure 3 illustrates the contribution level of different features before and after bias removal. As seen in this figure, the contribution of education in classifying cognitive impairment decreased after bias removal, while the relative contributions of the other features remained unchanged.

## Discussion

In this study, we developed fair machine learning classifiers, FaIRClocks capable of predicting cognitive impairment based on multiple cognitive measures with comparable performance of the patients with different educational levels. A compressed deep generative representation of the CDT was developed using a large-scale publicly available dataset for this purpose.

A comparison between the previously published RF-VAE latent space using 23,521 clocks with the one developed in this study using 54,027 publicly available unannotated clocks reveals a broad similarity between the two latent spaces. However, the current latent space captured a) the absence of digits and b) the rotation of the clockface as independent features. This is a result of the underlying differences in clock drawings present in the two datasets. ML classifiers performed better in all 3 tasks using the NHATS embedding compared to the previously published embedding. This shows that the NHATS embedding, which is developed using a larger number of clocks is decidedly better at capturing nuanced features, which were necessary for differentiating between low and high attention and memory composite Z-scores.

The comparison between the two clock drawing conditions, namely copy and command in the 3 tasks revealed that copy drawings are more predictive of attention deficits while command drawings are more predictive of memory deficits. Since MMSE is a composite attention and memory measure, the concatenated copy and command embeddings was the most predictive dataset for distinguishing between high and low MMSE scores. This finding is supported by previously existing literature in this domain ^3,31^.

The most significant finding of this study is the bias correction for patients with less than eight years of education in the MMSE and ATT-C prediction. While being tested separately, it was revealed that specificity = 0 and sensitivity = 1 in the uncorrected ML classifier for lower education patients. This finding shows that regardless of their cognitive condition, the original, uncorrected classifier consistently classified people with low education as cognitively impaired. Previous studies have reported the convergence between lack of education and cognitive impairment. Still, no studies have tested the sensitivity/specificity balance of commonly used CDT scoring mechanisms for low-education. Our analysis explicitly shows that an interpretable and generalizable deep representation of the CDT incurs the maximum false positive rate when predicting attention deficits or low MMSE in the unprivileged group. However, multiple reweighting methods available in the IBM AIF360 toolbox allowed these classifiers to be corrected such that they could achieve comparable sensitivity/specificity balance between low- and high-education patients, albeit with a marginal decrease in performance. Henceforth, this is the first study to evaluate the difficulty in measuring attention deficits through the CDT for people with less than eight years of education and mitigate the resulting bias using computational means.

Our study has several unique strengths, making it a pioneering work in bi-directional AI using cognitive science. Firstly, we developed the largest DCT model to date using clock drawings test images from NHATS and UF datasets for self-supervised pre-training of a generative model to represent the CDT and fine-tuning. Secondly, the generalized and interpretable latent space of the RF-VAE combined with retrospective SHAP analysis enabled us to investigate the relative importance of each latent dimension in predicting attention versus memory deficits in the unprivileged group versus the privileged group, thereby improving the interpretability and trustworthiness of our method. Thirdly, the ability of the RF-VAE latent space to compress a clock drawing into a ten-dimensional latent vector allowed us to use relatively smaller fine-tuning datasets to train lightweight task-specific ML classifiers downstream. Finally, the finding of high false-positive rates in attention deficit measurement for patients with lower education is a seminal finding in the cognitive domain which should stimulate further research into evaluating cognition for this often-underrepresented patient population. Furthermore, it should also stimulate discussion in the domain of neuropsychology towards developing a clock drawing test better suited for this patient population.

Our study has some limitations. Firstly, all clock drawings had to be resized to 64X64, which invariably caused obfuscation of some salient features (e.g., digit shape, arrowheads, ticks) used in traditional clock scoring methodologies. This explains the absence of these variables from the RF-VAE latent space. Secondly, the moderate performance of the classifiers shows that a lower-dimensional CDT representation alone is moderately predictive of attention/memory deficits. The addition of total clock scoring time, or total number of strokes improved the performance (Supplementary Table 3) which proves that additional features available from the dCDT contain non-redundant information over and above what might be available solely from the final output clock image. However, due to the absence of the total clock drawing time for the copy condition, in this current study, we were not able to incorporate such features. The extremely low proportion of low-education patients in our dataset (36/840, i.e., 4%) also restricts the generalizability of the findings. However, this further proves that collecting granular datasets of CDT and other cognitive measures for this vulnerable patient population is critical. Finally, the outcome variables were not associated with any gold standard diagnosis of cognitive impairment. Instead, they were based on population Z-scores calculated from neurocognitive assessments. This limits the clinical utility of our study.

In the future, we will continue to build more robust foundational generative models, such as the RF-VAE to represent the CDT and other visual cognitive assessments, such as the trail-making and maze completion tests. These foundational models will be iteratively updated using publicly available datasets whenever available. Using late-stage fusion, we will also incorporate relevant dCDT metrics in downstream classification tasks. Finally, this study will provide impetus for collecting datasets to evaluate cognition in lower educational groups and to specifically investigate the relevance of applying CDT in this population.

## Methods

### Patients

This study was conducted at The University of Florida (UF) and UF Health. Data were acquired by a federally funded investigation by The National Institutes of Health. The study followed the Declaration of Helsinki standards and UF’s Institutional Review Board-01 (IRB) approved this investigation, and the requirements for a written informed consent was waived by the IRB. Electronic Health record data were acquired through an IRB approved process which involved the deidentification of data provided through an honest data broker. This study is an extension of UF Health’s cognitive screener^32^ and is based on the implementation/creation of the Perioperative Cognitive Anesthesia Network (PeCAN^SM^) Clinic established in August of 2017 as part of UF Health’s Presurgical Center which older adults, aged 65 and older, that are believed to be at risk for cognitive change or confusion following surgery are referred to a preoperative neuropsychologist for a neurobehavioral examination.

Data for older adults seen at UF Health’s Presurgical Clinic and PeCAN^SM^ Clinic were retrospectively acquired during the period from January 3, 2018 through December 30, 2019. Using the clock drawing test and three-word recall described in Amini et al.^32^, patients were referred to the PeCAN^SM^ Clinic if patients missed one of the three words on recall, or had any one error out of ten on the clock drawing test. A neuropsychological protocol was administered by a licensed provider^33^. For the current investigation, data were extracted from the scored protocol and included the Mini Mental State Exam^34^, and also set of standardized neuropsychology measures^4,35^. A set of metrics were used to create composites for a memory domain (Hopkins Verbal Learning Test-Revised: list learning total immediate, delay, discrimination), and Attention domain (Letter F fluency, Wechsler Adult Intelligence Scale-Third Edition Digit Span Forwards, Backwards).

The development and test split ratio was set as 7:3 for the cognitive impairment classification based on three measures, and the patients in the training set and test set maintained the same in the three tasks. 5-fold cross-validation was further applied to the development set.

Another cohort of clock drawings used in this work is from the National Health and Aging Trends Study (NHATS) dataset, which conducts annual interviews with over 8,000 older adults in 11 rounds^36^.

### Clock drawings

Clock drawing images used to train the RF-VAE encoder were collected from the publicly available dataset provided by the National Health and Aging Trends Study (NHATS), which spanned 11 rounds of data collection in total from 2011 to 2022 and involved 8,000 elderly patients from the United States.

### Relevance factor VAE encoder

**Figure 4.**
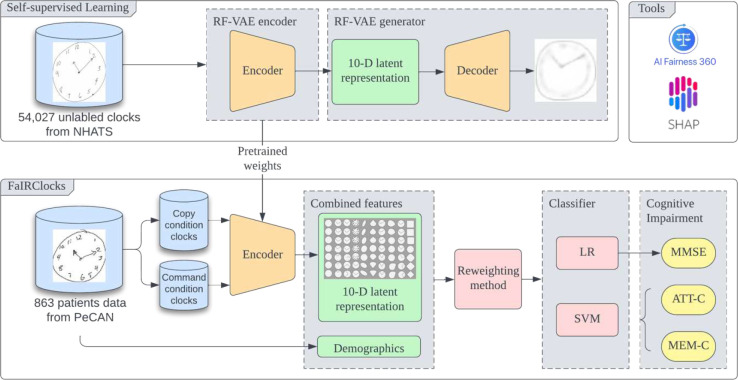
The overall conceptual flowchart of FaIRClocks is shown. The RF-VAE was pretrained on NHATS dataset. The trained RF-VAE encoder was used in the downstream classification pipeline, while the generator was not involved. The pretrained weights from the RF-VAE encoder were applied on the classification datasets within the FaIRClocks framework to generate 10-D latent representation for the command and copy conditions of the clock drawing test separately. These 10-D latent representations were combined with demographics information and fed to the classifier after reweighing using fairness functions from the AIF360 toolkit. Logistic regression produced the best classifier in predicting the MMSE score, while support vector machines created the best classifiers for predicting ATT-C and MEM-C scores.

We leveraged the RF-VAE, a self-supervised deep generative model to generate a 10-feature representation of CDT, which has been demonstrated to be effective in dementia screening as shown in our prior work ^22^. The overall workflow is shown in Figure 4. Instead of training the model on the private dataset, we pre-trained the RF-VAE encoder on the NHATS dataset which has 54,027 individual unlabeled clock drawings. The encoder was applied onto the clock drawing images collected from PeCAN program to generate a 10 dimensions latent space vector, while the generator was not included in the classification stage. We defined each of the 10 features as the following: Size, Vertical displacement of hands, Rotated clock, Digits, Ellipse, Obovate, Angle between hands, Side Bulge, Rotation of hand assembly, and Square-Rhomboid.

### Machine learning classifiers

Multiple machine learning classifiers are tested, including Logistic Regression (LR), Support Vector Machines (SVM), XGBoost, and a three fully connected layers neural network, aiming to find the best model for cognitive impairment classifying based on the three different measures. A binary threshold of −1 is set for both attention and memory z-scores, where values below −1 are classified as impaired, while values greater than or equal to −1 are classified as unimpaired. For MMSE total score, we follow the categorization approach proposed by Perneczky et al.^37^, and group class 1 (mild), class 2 (moderate), and class 3 (severe dementia) into a single category, resulting in a binary prediction task to detect whether the patient has cognitive impairment. The classifiers leverage the 10 dimensions of the RF-VAE features combined with basic demographics (age, sex, race, ethnicity, years of education and area deprivation index; ADI) as input, to generate binary outputs Y for each of the three tasks. In an additional follow-up analysis, we tested the improvement in classifier performance by including other clock variables such as total time to completion, and total number of strokes which were collected using smart paper and digital pen from Anoto corporation. Hyperparameters for all classifiers used a randomized grid search within a 5-fold cross-validation setting. Student’s T-Test with False Discovery Rate (FDR)=0.01 was used to calculate p-value inferring the significance.

### AI Fairness 360 toolkit

For further analysis, we considered the low education (<= 8 years of education) and the high education (> 8 years of education) patients in the test cohort and created performance indices for these two groups separately for the MMSE and ATT-C prediction. The total number of clocks drawn by low-education group was 36 and those drawn by high-education group were 804. Two fairness-related metrics were calculated to evaluate the model’s performance for different sub-groups. The 1-min(DI, 1/DI), where DI represents Disparate Impact which is a legal concept often used in the context of employment discrimination, as in Equation (1):

(1) $$DI=\frac{Pr\left(Y\;=\;1|D_{u}\right)}{Pr\left(Y\;=\;1|D_{p}\right)}$$

In Equation (1), Pr represents the probability, Y=1 means target value (have cognitive impairment or not), and $D_{u}$ and $D_{p}$ means unprivileged group and privileged group separately. The metric is designed to measure the balance between positive outcomes for different groups. Average odds different (AOD) shown in Equation (2) measures the average difference in the False Positive Rate (FPR, known as 1-specificity) and True Positive Rate (TPR, known as sensitivity) between two groups, which is also a good indicator for measuring sensitivity and specificity balance.

(2) $$AOD=\frac{\left(FPR_{D_{u}}\;-\;FPR_{D_{p}}\right)\;+\;\left(TPR_{D_{u}}\;-\;FPR_{D_{p}}\right)}{2}$$

To address the classifier bias (wherever it occurred) against the low-education group, we implemented the reweighting method, to reweight the samples belonging to low- versus high-education using the recommendations provided in previous studies^38^. A weight W was assigned to each sample in the dataset based on its demographic attributes and favorable target. For the favorable target Y=1 (cognitive impairment), we have weights set as $W_{D_{low},Y=1}=\frac{P(Y=1)}{P\left(Y=1|D_{low}\right)}$, and $W_{D_{high},Y=1}=\frac{P(Y=1)}{P\left(Y=1|D_{high}\right)}$ separately, where $D_{low}$ and $D_{high}$ are the sub-groups for different education levels. The weighted dataset will be the new input of the model.

## Figures and Tables

**Table 1.** Demographics of the fine-tuning dataset.

Dataset	Number of patients	Mean age (SD)	Mean education (SD)	% of female	% of Caucasian	Mean Target value
MMSE	840	75 (7)	13 (3)	50	80	26
ATT-C	731	75 (6)	13 (3)	51	82	−0.34
MEM-C	731	75 (7)	13 (3)	50	81	−1.02

Abbreviations. MMSE - mini mental state examination score, ATT-C - attention composite Z-score, MEM-C - memory composite Z-score

**Table 2.** Classification performance for MMSE prediction using Linear Regression.

Task	Dataset (Train:Test)	Fairness	AUROC (95% CI.)	F1-score (95% CI.)	Sensitivity (95% CI.)	Specificity (95% CI.)	Precision (95\% CI.)	1-min(DI, 1/DI) ↓	AOD ↓
MMSE	Copy (587:251)	-	0.73 (0.67–0.78)	0.47 (0.41–0.54)	0.60 (0.54–0.66)	0.72 (0.66–0.77)	0.39 (0.33–0.45)	0.48	0.37
		√	0.69 (0.63–0.74)	0.42 (0.36–0.48)	0.53 (0.47–0.60)	0.70 (0.64–0.76)	0.35 (0.29–0.41)	0.09	−0.19
	Command (587:252)	-	0.67 (0.62–0.73)	0.42 (0.36–0.48)	0.57 (0.51–0.63)	0.65 (0.60–0.71)	0.33 (0.27–0.39)	0.49	0.41
		√	0.64 (0.58–0.70)	0.41 (0.35–0.47)	0.55 (0.49–0.61)	0.65 (0.60–0.71)	0.32 (0.27–0.38)	0.21	0.05
	Copy+Comma nd (1174:503)	-	0.70 (0.66–0.74)	0.47 (0.43–0.51)	0.61 (0.57–0.65)	0.70 (0.66–0.74)	0.38 (0.34–0.42)	0.56	0.47
		√	0.66 (0.62–0.70)	0.44 (0.39–0.48)	0.57 (0.53–0.61)	0.69 (0.65–0.73)	0.35 (0.31–0.40)	0.05	−0.12

Abbreviations. MMSE - mini mental state examination score, AUROC - area under the receiver operating curve, C.I - confidence interval, DI - disparate impact, AOD – average odds different

Binarizing threshold. MMSE - scores of 0 are categorized as class 0, while scores of 1, 2, and 3 are categorized as class 1

**Table 3.** Classification performance for ATT-C and MEM-C prediction using SVM.

Task	Dataset (Train:Test)	Fairness	AUROC (95% CI.)	F1-score (95% CI.)	Sensitivity (95% CI.)	Specificity (95% CI.)	Precision (95\% CI.)	1-min(DI, 1/DI) ↓	AOD ↓
ATT-C	Copy (511:218)	-	0.69 (0.60–0.78)	0.35 (0.26–0.47)	0.50 (0.36–0.63)	0.71 (0.65–0.78)	0.28 (0.19–0.40)	0.50	0.39
		√	0.68 (0.59–0.76)	0.33 (0.23–0.45)	0.44 (0.31–0.58)	0.71 (0.65–0.78)	0.26 (0.17–0.38)	0.30	0.08
	Command (510:220)	-	0.68 (0.58–0.75)	0.36 (0.22–0.45)	0.50 (0.34–0.66)	0.69 (0.62–0.75)	0.28 (0.16–0.37)	0.46	0.37
		√	0.67 (0.59–0.77)	0.38 (0.27–0.51)	0.57 (0.42–0.74)	0.67 (0.60–0.74)	0.29 (0.20–0.40)	0.26	0.09
	Copy+Comma nd (1021:438)	-	0.71 (0.64–0.75)	0.41 (0.34–0.48)	0.57 (0.48–0.68)	0.71 (0.67–0.76)	0.31 (0.26–0.39)	0.52	0.43
		√	0.69 (0.63–0.74)	0.39 (0.31–0.45)	0.59 (0.48–0.68)	0.67 (0.62–0.72)	0.29 (0.23–0.36)	0.27	0.06
MEM-C	Copy (509:220)	-	0.68 (0.62–0.75)	0.67 (0.61–0.73)	0.73 (0.67–0.79)	0.63 (0.56–0.69)	0.62 (0.55–0.68)	0.38	−0.19
		√	0.68 (0.62–0.74)	0.67 (0.61–0.73)	0.73 (0.67–0.79)	0.63 (0.56–0.69)	0.62 (0.55–0.68)	0.39	−0.19
	Command (510:220)	-	0.70 (0.64–0.76)	0.67 (0.60–0.73)	0.73 (0.67–0.79)	0.63 (0.56–0.69)	0.62 (0.55–0.68)	0.39	−0.19
		√	0.70 (0.64–0.76)	0.67 (0.61–0.73)	0.73 (0.67–0.79)	0.63 (0.56–0.69)	0.62 (0.55–0.68)	0.39	−0.19
	Copy+Comma nd (1019:440)	-	0.67 (0.62–0.71)	0.61 (0.56–0.65)	0.60 (0.55–0.65)	0.68 (0.64–0.73)	0.61 (0.57–0.66)	0.05	−0.01
		√	0.67 (0.62–0.71)	0.61 (0.57–0.66)	0.60 (0.56–0.65)	0.68 (0.65–0.73)	0.61 (0.58–0.67)	0.26	−0.09

Abbreviations. ATT-C - attention composite Z-score, MEM-C - memory composite Z- score, AUROC - area under the receiver operating curve, C.I - confidence interval, DI - disparate impact, AOD - average odds different

Binarizing threshold. ATT, MEM - values greater than or equal to -1 are categorized as class 0, while values less than -1 are categorized as class 1.

## Data Availability

Datasets are available upon reasonable request. All dataset related queries should be directed to Dr. Catherine Price (cep23@phhp.ufl.edu). Reasonable requests will be reviewed to monitor compliance with the concerned authorities- National Institute of Health (NIH) and the Institutional Review Board (IRB).
